# Asynchronous vs didactic education: it’s too early to throw in the towel on tradition

**DOI:** 10.1186/1472-6920-13-105

**Published:** 2013-08-08

**Authors:** Jaime Jordan, Azadeh Jalali, Samuel Clarke, Pamela Dyne, Tahlia Spector, Wendy Coates

**Affiliations:** 1 Department of Emergency Medicine, Harbor-UCLA Medical Center, 1000 W. Carson St., Box 21, Torrance 90509-2910, CA, USA; 2 David Geffen School of Medicine at University of California, 10833 Le Conte Avenue, Los Angeles, CA 90095, USA; 3 Department of Emergency Medicine, University of California, Davis Medical Center, 2315 Stockton Boulevard, Sacramento, CA 95817, USA; 4 Department of Emergency Medicine, Olive View-UCLA Medical Center, 14445 Olive View Drive, Sylmar, CA 91342, USA; 5 Los Angeles Biomedical Research Center at Harbor-UCLA, Torrance, USA

**Keywords:** Medical student education, Emergency medicine, Computer based education, Asynchronous learning

## Abstract

**Background:**

Asynchronous, computer based instruction is cost effective, allows self-directed pacing and review, and addresses preferences of millennial learners. Current research suggests there is no significant difference in learning compared to traditional classroom instruction. Data are limited for novice learners in emergency medicine. The objective of this study was to compare asynchronous, computer-based instruction with traditional didactics for senior medical students during a week-long intensive course in acute care. We hypothesized both modalities would be equivalent.

**Methods:**

This was a prospective observational quasi-experimental study of 4th year medical students who were novice learners with minimal prior exposure to curricular elements. We assessed baseline knowledge with an objective pre-test. The curriculum was delivered in either traditional lecture format (shock, acute abdomen, dyspnea, field trauma) or via asynchronous, computer-based modules (chest pain, EKG interpretation, pain management, trauma). An interactive review covering all topics was followed by a post-test. Knowledge retention was measured after 10 weeks. Pre and post-test items were written by a panel of medical educators and validated with a reference group of learners. Mean scores were analyzed using dependent *t*-test and attitudes were assessed by a 5-point Likert scale.

**Results:**

44 of 48 students completed the protocol. Students initially acquired more knowledge from didactic education as demonstrated by mean gain scores (didactic: 28.39% ± 18.06; asynchronous 9.93% ± 23.22). Mean difference between didactic and asynchronous = 18.45% with 95% CI [10.40 to 26.50]; p = 0.0001. Retention testing demonstrated similar knowledge attrition: mean gain scores −14.94% (didactic); -17.61% (asynchronous), which was not significantly different: 2.68% ± 20.85, 95% CI [−3.66 to 9.02], p = 0.399. The attitudinal survey revealed that 60.4% of students believed the asynchronous modules were educational and 95.8% enjoyed the flexibility of the method. 39.6% of students preferred asynchronous education for required didactics; 37.5% were neutral; 23% preferred traditional lectures.

**Conclusions:**

Asynchronous, computer-based instruction was not equivalent to traditional didactics for novice learners of acute care topics. Interactive, standard didactic education was valuable. Retention rates were similar between instructional methods. Students had mixed attitudes toward asynchronous learning but enjoyed the flexibility. We urge caution in trading in traditional didactic lectures in favor of asynchronous education for novice learners in acute care.

## Background

Methods of education continually evolve to meet the ever-changing needs of instructors and learners. Asynchronous, computer-based instruction has become more prevalent in recent years. Asynchronous learning is a student centered instructional method where interactions between teachers and learners occur independent of place and time [1]. This type of learning holds the potential for increased cost-effectiveness, greater flexibility, self-directed pacing and review, and improved efficiency of educator resources [2,3]. These potential benefits particularly apply to medical education where new discoveries are made daily, placing increasing demands on educators and students to share knowledge despite increased regulations of duty hours and costs. In post-graduate training programs, the prospect of including asynchronous learning modules to meet the conference attendance requirements set forth by the Accreditation Council for Graduate Medical Education (ACGME) [4] is highly desirable. Additionally, in 2008, a report issued by the Macy Foundation highlighted the need for individual practitioners to monitor and address their own learning to keep up with changes in medical practice [5]. Asynchronous education can provide this opportunity, thus has large appeal in the medical profession.

Most research surrounding computer based learning to date suggest that there is no significant difference between traditional classroom-based and asynchronous, computer-based instruction. Randomized controlled trials looking at the effectiveness of an asynchronous, computer based course versus traditional classroom teaching of evidence based medicine found that computer-based learning was as effective at increasing a student’s knowledge as traditional classroom teaching [6,7]. A 2008 meta-analysis regarding internet based learning in the health professions utilizing data from 76 studies concluded that there was no significant difference in effectiveness between traditional and internet based methods [8]. Other studies have been performed in infection control [9], neuroanatomy [10], and respiratory medicine [11], which showed a similar effect between classroom and asynchronous, computer-based instruction. There are limited data on this comparison in acute care or emergency medicine (EM); however, a few studies looking at the instruction of emergency procedures via an online asynchronous format have yielded positive results [12,13].

At the David Geffen School of Medicine at the University of California, Los Angeles, fourth year students self-select and enroll in one of five colleges based on their future career choice or interest in the unique curricular offerings of an individual college. The Acute Care College (ACC) is typically comprised of students who plan to specialize in Anesthesiology, EM, or Critical Care. Students of the ACC are required to participate in an intensive, weeklong educational experience at the beginning of the fourth year. This course consists of didactic lectures, small-group interactive activities, procedural training on unembalmed cadavers, full-scale human simulation of critical patient management scenarios, and career development sessions. Students in previous years have asked repeatedly for more hands-on time in the simulation, cadaver, and procedural labs, but educators have struggled to increase this component without compromising time spent on the acute care core content that is essential to the students’ knowledge base to prepare them for both the interactive sessions and eventual patient management. In prior years, this standard curriculum was delivered in classroom lecture format at the beginning of each day of the course. To address the desire to expand the smaller group formats, we offered asynchronous, computer-based instruction to deliver half of the core course content to our novice learners, thus freeing up more time for hands-on and small group experiences. We hypothesized that there would be no significant difference between the two instructional methods. The objectives of our study were to: (1) assess if senior medical students improve their medical knowledge with asynchronous, computer-based instruction and traditional didactic lectures to the same level during a one-week required course in acute care; and (2) explore students’ attitudes towards asynchronous and didactic instruction.

## Methods

### Study setting and participants

All fourth-year medical students enrolled in the 2011–2012 ACC Foundations Course at the David Geffen School of Medicine at UCLA participated. This study was certified as exempt by the Institutional Review Board from the David Geffen School of Medicine at UCLA Office for Protection of Human Subjects.

### Study design

This was a prospective, observational, quasi-experimental curricular evaluation study. Pre- and post-test items were developed and revised by the authors (all were course faculty). Post-test questions differed from the pre-test questions to minimize recollection bias, but covered the same topics. Items were randomly assigned, by our non-physician study coordinator, to be on either the pre- or post-test. Both knowledge tests were carefully blueprinted to ensure each form contained an equal number of items of critical topics from each module, ensuring content validity. Pre- and post-tests were found to be of comparable difficulty by utilizing a separate group of reference learners, emergency medicine interns, whose mean pre-test score was 55.83% ± 15.51 and mean post-test score was 58.33% ± 11.68. This difference was not significant with a mean difference of −2.5% [−17.67 to 12.67]; p = 0.76.

Further in depth analysis of the reference group found that there was not a significant difference in performance on pre-test questions covering asynchronous content and pre-test questions covering didactic content with mean scores of 60.72% ± 19.84 and 53.13% ± 12.94 respectively with a mean difference of 7.60% [−5.07 to 20.25]; p = 0.20. There was also not a significant difference in performance of the reference group on post-test questions covering asynchronous and didactic content with mean scores of 65.63 ±17.36 and 48.21 ± 15.15 respectively with a mean difference of 17.41% [−0.66 to 35.48]; p = 0.57. Lastly the reference group did not show a significant difference in performance between pre- and post-test questions covering asynchronous content (mean scores of 60.72 ± 19.84 and 65.63 ± 17.40 respectively, mean difference of 4.91% [−26.54 to 16.72]; p = 0.61) or didactic content (mean score of 53.13 ± 12.94 and 48.21 ± 15.15 respectively, mean difference of −4.91% [−12.53 to 22.35]; p = 0.53). This comprehensive analysis suggests that pre- and post-tests were of similar difficulty for both asynchronous and didactic content.

**Figure 1:**
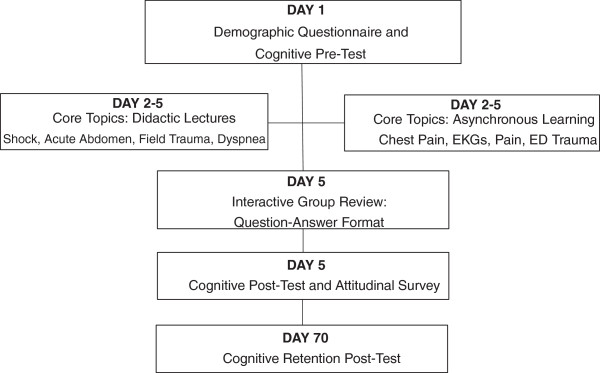
Study flowchart.

Figure 1 depicts the flow of our study. On Day 1, each participant provided demographic data (age, gender, and intended specialty), followed by a knowledge pre-test comprised of multiple choice single answer questions about the core didactic topics (shock, acute abdomen, dyspnea, trauma, chest pain, EKG interpretation, and pain management).

The standard curriculum on these topics was delivered in either traditional classroom lecture format (shock, acute abdomen, dyspnea, ED trauma) or via asynchronous, computer-based modules (chest pain, EKG interpretation, pain management, field trauma). Selection of mode of teaching was based on the desire to vary content and to accommodate professor availability. To the best of our knowledge there was nothing inherent in the topics that would make them more amenable to either method of delivery. The professors conducting the real-time sessions were the usual lecturers for the course and welcomed audience participation. All had earned excellent to outstanding evaluations by students in prior years. Each lecture was 45 minutes in duration and was scheduled as the first activity of the morning.

The asynchronous, computer-based modules consisted of two digitally recorded podcast lectures (chest pain and acute pain management) by the same faculty who gave them annually as “live” sessions in previous years and who were also highly rated by prior students. These lectures were assigned to this modality based on faculty willingness to convert their usual lecture to an asynchronous format. Both specifically highlighted concerns that had been raised consistently by students in prior years. The third module (field trauma) was a video of trauma cases and debriefings that was shown in previous years as the didactic session, in which the creating professor was present to answer questions and assure compliance. The final asynchronous module (EKG interpretation) was prepared specifically for asynchronous education. It consisted of examples of EKGs with detailed analysis and explanations. This was similar to the format used when this topic had been delivered in the large group format.

All four asynchronous, computer-based modules were uploaded to the university’s educational website which is familiar to all students. The website can be accessed remotely, allowing students to view the modules on their own time in the location of their choosing, including their own personal or home computers. Additionally, students were allocated one daily block of unscheduled time during the week where they had no structured activities and could choose to use the time to complete the asynchronous modules, ask for clarification from course faculty, meet with mentors, or tend to personal matters. The medical school’s computer learning laboratory was reserved for their use throughout the course, and they were also entitled to use university computers after hours. The length of time spent on the asynchronous, computer-based modules was self-directed and unlimited, and students had the option to go back and review the material at any time. While students were allowed to perform asynchronous learning in teams, each had to log on to document enrollment in each module to receive course credit.

On the final day (Day 5), faculty conducted a 1-hour group interactive, question-answer review session equally covering both asynchronous and traditional topics. The purpose of this review was to ensure that everyone had an opportunity to clarify concepts and fill in gaps in knowledge on all subject matter. At the end of Day 5, students took a multiple choice post-test to assess their knowledge gain and completed a five-point Likert scale questionnaire to assess attitudes toward asynchronous and didactic instruction. Ten weeks later, students again completed the multiple choice post-test to assess knowledge retention, during a regularly scheduled educational meeting.

### Data analysis

We calculated the percentage of correctly answered questions for each instructional method and computed a gain score by subtracting pre-test scores from post-test scores. We compared instruction methods using a dependent *t*-test of mean gain scores. Data were analyzed with IBM SPSS version 20 (IBM Software group, Chicago, IL).

## Results

All 48 students enrolled in the course consented to participate in the study. 44 of 48 completed the study protocol. Four were absent during the administration of the 10-week retention test and so their data were not included in the analysis. Demographic characteristics are displayed in Table 1.

**Table 1:** Student demographics

	**Total n = 44**
Mean age (years)	25.8
Female	16
Male	28
Intended specialty:	
Critical care	22
Emergency medicine	15
Anesthesia	7

Results reported as *n* or years.

Mean gain in knowledge was 28.39% ± 18.06 for didactic instruction and 9.93% ± 23.22 for asynchronous instruction. A two-tailed dependent *t*-test revealed a statistically significant mean difference between didactic and asynchronous modalities of 18.46% with 95% CI [10.40 to 26.50]; p = 0.0001. Retention testing after 10 weeks demonstrated similar knowledge attrition for both groups. Mean gain scores (relative to post scores) were −14.94% (didactic); -17.61% (asynchronous). This was not significantly different, with a mean difference of 2.67% ± 20.85, 95% CI [−3.66 to 9.02], p = 0.399. Mean scores for the retention test in the asynchronous group were lower than pre-test scores, 51.99% ± 13.46 and 62% ±15.69 respectively. This difference was statistically significant with a mean difference of −10.02% ± 15.48, 95% CI [−14.73 to −5.32]; p = 0. Mean gain scores are depicted in Table 2. Mean scores for pre, post, and retention testing are depicted in Table 3. There was a significant difference in pre-test scores for asynchronous and didactic groups with mean scores of 62% ± 15.69 and 39.75% ± 13.29 respectively with mean difference of 22.24% ± 22.06, 95% CI [15.53 to 28.95]; p = 0.

**Table 2:** Mean gain scores

	**Pre test to post test % ± SD**	**Post test to retention test % ± SD**
Didactic	28.39 ± 18.06	−14.94 ± 18.73
Asynchronous	9.93 ± 23.22	−17.61 ± 17.12
Mean difference% ± SD	18.45 ± 27.72	2.68 ± 20.85
95% CI	[10.40 to 26.50]	[−3.66 to 9.02]
p value	p = 0.0001	p = 0.399

**Table 3:** Mean test scores

	**Pre test % ± SD**	**Post test % ± SD**	**Retention test % ± SD**
Didactic	39.75 ± 13.29	67.86 ± 11.99	52.92 ± 18.89
Asynchronous	62 ± 15.69	69.63 ± 15.10	51.99 ± 13.46

**Figure 2:**
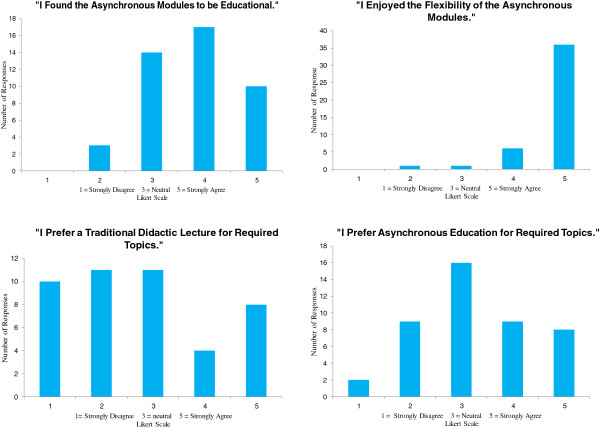
Results of attitudinal survey.

Students had mixed attitudes towards asynchronous education (Figure 2). Students uniformly (95.8%) enjoyed the flexibility afforded by asynchronous learning however, only 60.4% believed the asynchronous modules were educational. When asked which type of learning they preferred, 39.6% indicated that asynchronous education was preferable for required didactics, 37.5% were neutral, and 23% preferred traditional lectures.

## Discussion

The results of this study suggest that asynchronous, computer-based instruction is not equivalent to didactic instruction of an acute care curriculum for novice learners. In a rapidly changing educational climate where increasing constraints are placed on funding and time, asynchronous, computer based instruction seems like an exciting alternative; however, like any new method, it lacks a thorough evidence based assessment. Asynchronous learning requires a substantial investment of resources initially for product design, implementation, and monitoring, but offers long term benefits of saving instructor time and reducing logistical planning for classroom space in subsequent years. Additionally, student learning may be facilitated by allowing students to learn at a time, place, and setting convenient for them. Asynchronous learning can also free up time in a curriculum for more hands on learning of topics that must be conducted in a face-to-face manner. Despite these stated benefits, asynchronous learning may not be worth the investment if we do not find at least equivalency with current teaching methods. It is prudent to be wary of investing in a program that has not been fully evaluated.

Our results contrast with much of the current literature that generally has found that asynchronous and didactic education are equivalent. We hypothesize that there are several factors that may account for this. First, students had a higher baseline knowledge of the asynchronous topics, as evidenced by a higher mean pre-test score. This finding was not demonstrated in the reference group. Therefore, the lack of gain seen with this method may have been due partially to a ceiling effect. This higher baseline knowledge may be due to prior education, though these students had received only minimal exposure to the studied content. Larger, multi-institution studies may elucidate whether this finding is consistent.

Additionally, while didactic education is considered to be a one-way transmission of material from teacher to learner, we cannot overlook the possibility of meaningful interaction between experts and learners during live lectures. This type of interaction, which allows for immediate clarification of concepts and extension of knowledge, may be particularly important for novice learners who have relatively little exposure to the subject matter, such as our study population. Most rigorous studies on design factors for asynchronous “distance learning” education can be found in the literature describing undergraduate (college) education, and have emphasized the importance of interaction amongst the peer learners and between the instructors and students, that focuses on course content and learning issues. This “community of inquiry” has a profound impact on learning outcomes and satisfaction [14,15]. Increased interaction within asynchronous modules, amongst peers and between learners and instructors via a discussion board may yield more positive results for asynchronous learning, as it did in a study of EM house officers [16]. In our model, we believed that we had provided students with the opportunity to seek feedback and ask questions by carving out a designated time during their individual schedules for each day. In past years, we noted that some students felt frustrated by the rapid pace of traditional didactic learning without an opportunity to clarify points individually. Some complained that the lectures had moved forward too quickly and that they wished they could have repeated the basics before attempting the more difficult material. Others indicated a need for unscheduled time during the business day to attend to matters such as mentor meetings and personal issues.

Many studies on asynchronous instruction in medical education and healthcare focused on a single topic [7,9-11,13]. Our study, while aimed at a single learner group, sought to impart information across a diverse range of topics in acute care. We believe that the required use of an interactive supplement to the independent learning modules in the form of specific face-to-face encounters or mandatory online teacher-moderated discussion boards would mitigate the difficulties inherent in mastering new information. Successful university programs cited this as a key element when their asynchronous learning course had a broad focus [14,15]. It is likely that frequent knowledge checks and student accountability would create a positive learning condition, and could provide an avenue for clarification that might otherwise seem daunting for a novice learner.

Our results may also indicate a mismatch between educational methods and learner needs. While asynchronous instruction does appeal to today’s millennial learner’s preference for flexibility, use of technology, and audio-visual stimulation, it also places a significant amount of responsibility and need for independent monitoring on the learner. This self-directed approach may be more difficult for the millennial learner who typically has had a high level of parental and teacher involvement. These learners thrive on immediate delivery of knowledge and formative feedback to maintain focus and enhance learning [17]. In modules such as those in our course, this could come in the form of frequent formative quizzes and “hard stops” that would require the learner to demonstrate understanding of information before moving on to the next step. Ready access to others on a discussion board might serve a similar purpose.

Students’ attitudes may also have contributed to our results. No educational method can succeed without a critical amount of learner “buy-in” and, as demonstrated by our attitudinal assessment, this study population, while generally favorable, had mixed opinions regarding asynchronous compared to didactic instruction. Current literature on this topic does not always include learner attitudes towards various methods and whether they correlate with outcomes. To ensure success of asynchronous instruction, curriculum developers must identify why students may not have positive attitudes towards this modality and create solutions to address these barriers.

Asynchronous learning may be a tool that is better used as a complement to traditional education rather than a replacement, especially in the setting of EM. Blended learning that combines interactive didactics with asynchronous modules may be a successful method, capitalizing on the strengths of both techniques and minimizing the weaknesses [18]. The few studies in the field of EM support more of a blended model rather than favoring one or the other [19,20].

Lastly, though our study suggests that asynchronous and didactic education may not be equivalent for knowledge acquisition, we did see similar knowledge attrition after a period of time in both groups so that we cannot favor one method over the other in terms of retention of knowledge. Interestingly, mean retention scores for the asynchronous group were lower than mean pre-test scores. We did not find a significant difference between test items in our reference group. On the surface, it seems counterintuitive that students would lose more knowledge than they started out with. We postulate that students may have faced some degree of “information overload” given the intense knowledge acquisition across several different topics in a relatively short time frame. Additionally, students who were complete novices may have been able to answer the questions from a simpler perspective. As they acquired more knowledge during their clinical encounters and extended their experience, the objective test items may have evoked a more complicated thought process that extended beyond the straightforward answers that were more obvious when the subject material represented the majority of their understanding at the beginning of the academic year. Improving knowledge retention through various instructional methods is another area that warrants further research.

### Limitations and future directions

This study took place at one institution, so the results may be difficult to generalize. It is particularly difficult to perform a randomized, controlled study in the educational setting. Because this was a required course, we felt it would be unethical to randomize students to different experiences. Additionally, it is possible that our interactive review on Day 5 could have accounted for the lack of difference between groups in the post-test. It may have been more telling if we had conducted the post-test prior to this session. It is also important to note that the term “asynchronous” applies to an enormous variety of educational modalities - from podcasts to interactive games. We urge caution in generalizing our results to all formats of asynchronous instruction. Our curriculum included a variety of teaching modalities. It is likely that each student could find something appealing in this mix, however, the superiority of any one modality could not be determined with this approach. We felt such a varied curriculum was more representative of our existing course and chose to evaluate it as a whole.

Despite these limitations, our study does provide significant results that should encourage the medical education community to further examine the merits of traditional lectures and asynchronous educational methods. We urge educators to select the appropriate method based on their learners’ needs. Asynchronous, computer-based instruction still has the potential for significant value as a curricular component. However, we caution against “jumping on the asynchronous band-wagon” and closing the door on traditional methods that have withstood the test of time. There are still many questions left unanswered. More research is required, perhaps beginning with a qualitative exploration, to define the value and application of asynchronous education in the setting of EM and acute care, particularly when the learners are novices. Proper attention in selecting effective asynchronous topics, methods, and targeting appropriate learner groups should be further elucidated. Inclusion of built-in interaction with peers and faculty in a virtual or physical environment may enhance the utility of asynchronous instruction.

## Conclusion

Asynchronous, computer-based modules alone were not equivalent to traditional lectures for novice learners of acute care topics. Didactic education was valuable in this setting for immediate mastery of content, although retention rates were similar between methodologies. Students had mixed attitudes towards asynchronous education. We urge caution in trading in traditional didactic lectures in favor of asynchronous methods for novice learners in EM and acute care.

## Abbreviations

ACGME: Accreditation council for graduate medical education; EM: Emergency medicine; ACC: Acute care college.

## Competing interests

The authors declare that they have no competing interests.

## Authors’ contributions

JJ is the principal investigator for this study and participated in study design, served as core faculty, wrote test items, and drafted the manuscript. AJ is responsible for maintaining and analyzing data for the study. SC served as core faculty wrote test items, and contributed to the manuscript. PD served as core faculty, wrote test items, and contributed to the manuscript. TS served as core faculty and contributed to the manuscript. WC supervised all aspects of the study and edited the manuscript. All authors read and approved the final manuscript.

## Authors’ information

JJ is a fellow in Medical Education at Harbor-UCLA Department of Emergency Medicine and Director of Asynchronous Learning for the Acute Care College at UCLA School of Medicine. AJ is a research associate and the coordinator for the Acute Care College at UCLA School of Medicine. SC is assistant professor of Emergency Medicine at University of California, Davis. During this project, he was a Medical Education fellow at Harbor-UCLA Emergency Medicine and Director of Simulation Education for the Acute Care College at UCLA. PD is Professor of Medicine at UCLA School of Medicine and Director of Preclinical Education for the Acute Care College. TS is Assistant Professor of Medicine at UCLA School of Medicine and Director of Continuing Education for the Acute Care College. WC is a Professor of Medicine and Chair of the Acute Care College at UCLA School of Medicine; Director of Medical Education, and Director of the Fellowship in Medical Education at Harbor-UCLA Department of Emergency Medicine.

## Pre-publication history

The pre-publication history for this paper can be accessed here:

http://www.biomedcentral.com/1472-6920/13/105/prepub

## Presentations

1. Oral Research Presentation at the Plenary Session of the Academic Assembly: Council of Residency Directors (CORD), April 2012, Atlanta, GA;

2. Oral Research Presentation at the Society: Academic Emergency Medicine (SAEM) Western Regional Forum, March 2012, Las Vegas, NV;

3. Moderated poster: SAEM Annual Meeting, May 2012, Chicago, IL.
